# Hypermethylated DNA, a circulating biomarker for colorectal cancer detection

**DOI:** 10.1371/journal.pone.0180809

**Published:** 2017-07-10

**Authors:** Simon Ladefoged Rasmussen, Henrik Bygum Krarup, Kåre Gotschalck Sunesen, Martin Berg Johansen, Mogens Tornby Stender, Inge Søkilde Pedersen, Poul Henning Madsen, Ole Thorlacius-Ussing

**Affiliations:** 1 Department of Gastrointestinal Surgery, Aalborg University Hospital, Aalborg, Denmark; 2 Clinical Cancer Research Center, Aalborg University Hospital, Aalborg, Denmark; 3 Department of Clinical Medicine, Aalborg University, Aalborg, Denmark; 4 Section of Molecular Diagnostics, Clinical Biochemistry, Aalborg University Hospital, Aalborg, Denmark; 5 Unit of Clinical Biostatistics, Aalborg University Hospital, Aalborg, Denmark; Institut de genomique, FRANCE

## Abstract

**Background:**

Colorectal cancer (CRC) is one of the most common cancers in the western world. Screening is an efficient method of reducing cancer-related mortality. Molecular biomarkers for cancer in general and CRC in particular have been proposed, and hypermethylated DNA from stool or blood samples are already implemented as biomarkers for CRC screening.

We aimed to evaluate the performance of proven hypermethylated DNA promoter regions as plasma based biomarkers for CRC detection.

**Methods:**

We conducted a cross-sectional case-control study of 193 CRC patients and 102 colonoscopy-verified healthy controls. Using methylation specific polymerase chain reaction, we evaluated 30 DNA promoter regions previously found to be CRC specific. We used multivariable logistic regression with stepwise backwards selection, and subsequent *leave-pair-out cross validation*, to calculate the optimism corrected area under the receiver operating characteristics curve (AUC) for all stage as well as early stage CRC.

**Results:**

None of the individual DNA promoter regions provided an overall sensitivity above 30% at a reasonable specificity. However, seven hypermethylated promoter regions (*ALX4*, *BMP3*, *NPTX2*, *RARB*, *SDC2*, *SEPT9*, and *VIM*) along with the covariates sex and age yielded an optimism corrected AUC of 0.86 for all stage CRC and 0.85 for early stage CRC. Overall sensitivity for CRC detection was 90.7% at 72.5% specificity using a cut point value of 0.5.

**Conclusions:**

Individual hypermethylated DNA promoter regions have limited value as CRC screening markers. However, a panel of seven hypermethylated promoter regions show great promise as a model for CRC detection.

## Introduction

Colorectal cancer (CRC) is one of the most common cancers, reaching annual incidence rates of >1.3 million cases worldwide [1]. Ensuring survival of CRC patients relies on detection at early stages, which can be achieved through population-based screening [2,3]. The current screening program in Denmark includes an immunochemical faecal occult blood test (iFOBT) followed by a colonoscopy if iFOBT is positive.[3] However, patient adherence to current screening programs is limited by low completion rates with great differences between ethnic groups [4]. This necessitates the development of other methods for early CRC detection in order to ensure patient survival. Cell-free nucleic acids are currently of great interest as blood-based biomarkers for cancer, as these might be more specific for individual cancer types than protein-based biomarkers [5]. Methylation occurs across the whole genome with the exception of gene promoter regions which are preferably hypomethylated. Hypermethylation of the gene promoter regions induces gene silencing in two ways: (i) inhibition of transcription factor binding, and (ii) binding of methyl-binding domain proteins, which enable chromatin modifications. DNA hypermethylation occurs early and often in the adenoma to carcinoma sequence promoting it as an ideal blood-based biomarker for CRC. This has led to the development of a commercially available blood test for DNA methylation analysing the promoter for the *SEPT9* gene [6]. However, evaluation of the *SEPT9* assay in a large-scale cross-sectional study revealed sub-optimal sensitivity and specificity (48.2% and 91.5% respectively) [7]. Other case-control studies analysing a larger panel of genes, have revealed more promising results, however, these studies are often small and lack well-defined control groups [8].

The aim of this study was to evaluate the performance of a panel of hypermethylated promoter regions in plasma for CRC detection, in order to enable blood-based CRC screening.

## Methods

### Study population

This was a cross-sectional study analysing the ability of plasma derived cell-free DNA hypermethylation to distinguish patients with CRC from a control group of patients suspected for but without CRC. Between 2003 and 2005 at The Department of Gastrointestinal Surgery, Aalborg University Hospital, a consecutive series of CRC patients were included to evaluate the correlation between coagulation status and CRC. The inclusion of the patients is described elsewhere [9]. All patients with diagnosed CRC and available blood samples at the time of inclusion were included in the current study. During the same study period, blood samples from patients referred for colonoscopy with symptoms of, but without CRC were also collected. These patients were of a similar age as the CRC patients, and constituted the control group.

We classified patient tumours according to the tumour, node, and metastasis (TNM) system, and staging was in accordance with the American Joint Committee on Cancer staging system (AJCC) 7^th^ Edition.

Written informed consent was obtained from all patients, and the initial study was approved by The North Denmark Region Committee on Health Research Ethics (N-20040067). The subsequent hypermethylation analysis was also approved by The North Denmark Region Committee on Health Research Ethics (N-20140064) and registered at ClinicalTrials.gov (NCT02928120).

### Outcome and predictor variables

We aimed to establish a multivariable prediction model for CRC detection using a panel of 30 promoter regions, previously evaluated in stool or blood, as biomarkers for CRC with varying sensitivities [10]. Thus, we defined the outcome variable as patients with/without CRC.

Along with the covariates sex and age, these 30 hypermethylated promoter regions were the potential predictor variables. A list of the gene names and their function is provided in the supplementary material (S1 Table).

### Blood sampling

Blood samples from the CRC patients were obtained at the time of diagnosis and before any kind of treatment. Blood samples from the control group were obtained prior to colonoscopy. Blood sampling was conducted in accordance with The European Concerted Action on Thrombosis (ECAT) procedures. All blood samples were centrifuged at 4°C for 20 minutes at 4000 rpm and the plasma samples were immediately stored at -80°C for future use. The period from sample acquisition to storage never exceeded two hours.

### Hypermethylation analysis

The method for DNA extraction and methylation analysis is based on a rapid bisulphite-treatment protocol described by Pedersen et al. (2012) [11]. A schematic overview of the experimental protocol is appended in the supplementary material (S1 Fig).

Plasma nucleic acids were extracted from 450–1000 μl plasma samples using The easyMag^TM^ platform (NucliSens^®^ [bioMérieux SA, France]) according to the manufacturers’ instructions. The purified nucleic acids were eluted in 35 μl elution buffer (NucliSens^®^ [bioMérieux SA, France]). Five μl DNA extract were used for quantitation. The remaining 30 μl was mixed with 60 μl deamination solution, deaminated for 10 minutes at 90°C, followed by a purification step (using the easyMag^TM^ platform) and lastly eluted in 25 μl 10 mM KOH. In order to expand the deaminated DNA, we conducted a first round polymerase chain reaction (PCR) using a mix of methylation specific outer primers for all the investigated promoter regions. The reaction buffer (25 μl) consisted of PCR buffer, 13 μM MgCl_2_, 0.6 mM dNTP (dTTP is replaced by dUTP), 250 nM of each outer primer, 1.5 U Taq polymerase (BIOTAQ^TM^ [Bioline^®^, Taunton, MA, USA]), and 0.3 U Cod Uracil-DNA Glycosylase (Cod UNG [ArcticZymes^®^, Tromsoe, Norway]) in order to limit the risk of contamination in the two step PCR. We distributed the first round reaction mix to individual 200 μl PCR tubes, which were incubated for five minutes at 37°C, followed by 95°C for five minutes, and cooled to room temperature. Thereafter, we added 25 μl of purified deamination product to each tube yielding a reaction volume of 50 μl. For the second PCR, we distributed 10 μl buffer containing 0.4 μM methylation specific inner primers and probes in 30 individual wells in a 96 well PCR plate. We added 10 μl of the first round PCR product to 710 μl preincubated reaction mix (37°C for five minutes and 95°C for 10 minutes) containing PCR buffer, 250 μM dNTP, 10 μM MgCl_2_, 8 U Taq polymerase (BIOTAQ^TM^ [Bioline^®^, Taunton, MA, USA]), and 0.8 Uracil-DNA Glycosylase (Invitrogen^®^, Waltham, MA, USA). Twenty μl of reaction mix was then added to each of the 30 wells to a final reaction volume of 30 μl.

All hypermethylation specific primer and probe sequences along with amplicon sizes are available in the supplementary material (S2 and S3 Tables).

### Statistical analysis

We handled the outcome variable (CRC/no CRC) and the potential predictor variables: hypermethylated DNA promoter regions (hypermethylated/not hypermethylated), CEA-levels (>/≤ 5 ng/ml, sex (male/female), and age (age>66/age≤66) as binary variables. This rendered the data suitable for logistic regression modelling. We used all the patients in the model development process and the subsequent cross-validation.

First, we calculated the median and range of the number of hypermethylated promoter regions and cell-free DNA concentrations according to CRC patients and controls. We compared the two patient groups using the non-parametric Wilcoxon-Mann-Whitney test. Secondly, using logistic regression, we performed a univariate screening of potential predictors according to the outcome variable at a significance level of p = 0.3. Thirdly, we constructed a multivariable logistic regression model, using stepwise backwards selection. Receiver operating characteristic (ROC) curves are presented and the area under the ROC curve (AUC) calculated. Interactions were evaluated between all the potential predictor variables included in the final model. All models were evaluated using Hosmer-Lemeshow’s goodness of fit test. Fourthly, we computed a Penalized regression model using Firth’s method with backwards selection, to assess the impact of separation issues on the model building process. Lastly, we performed internal validation using “Leave pair out cross validation” computing the optimism corrected AUC in order to evaluate the predictive performance of the final prediction model. The predictive performance of the final model was evaluated for all patients and after restriction to early-stage CRC patients (stage I-II).

We used STATA^®^ V.13.1 (StataCorp LP, TX, USA) for all statistical analyses.

## Results

### Study population

Review of the CRC patients led to the exclusion of twelve patients: seven with benign disease or non-colorectal cancer, three did not have any residual cancer after endoscopic resection, one patient initially refused surgery, and one patient did not provide informed consent. Five additional patients were excluded, because the reference gene could not be amplified during PCR analysis (S2 Fig). Review of the control patients lead to the exclusion of five patients with inflammatory bowel disease and 27 patients diagnosed with any kind of neoplastic disease in the years following inclusion. This left blood samples from 193 CRC patients and 102 controls without CRC. The remaining control group included 33 patients with resectable adenomas, none of which were of high-grade dysplasia. Patient characteristics are provided in Table 1.

**Table 1:** Patient characteristics.

	*Colorectal Cancer*		*Healthy Controls*	
N	193		102	
Age, mean (SD)	67.5	(11.5)	64.7	(14.2)
Sex, n (%)				
Male	119	(61.7)	55	(53.9)
Female	74	(38.3)	47	(46.1)
Smoke status, n (%)				
Never smoker	68	(35.7)	31	(30.4)
Current smoker	77	(39.9)	28	(27.5)
Previous smoker	43	(22.3)	24	(23.5)
Unknown	5	(2.6)	19	(18.6)
CEA-levels				
≤ 5 ng/ml	141	(73.1)	91	(89.2)
> 5 ng/ml	52	(26.9)	11	(10.8)
Tumour, n (%)				
T1	3	(1.6)	-	-
T2	30	(15.5)	-	-
T3	120	(62.2)	-	-
T4	34	(17.6)	-	-
T-unknown	6	(3.1)	-	-
Node, n (%)				
N0	121	(62.7)	-	-
N1	38	(19.7)	-	-
N2	28	(14.5)	-	-
N-unknown	6	(3.1)	-	-
Metastasis, n (%)				
M0	159	(82.4)	-	-
M1	34	(17.6)	-	-

Note. The number (N) of patients in each patient group along with the mean age and standard deviations (SD) in each group. The number (n) and percentages (%) of patients according to sex, smoke status and carcinoembryonic antigen levels along with the colorectal cancer patients according to the tumour, node, and metastasis (TNM) classification system is also presented.

### Model development

**Fig 1:**
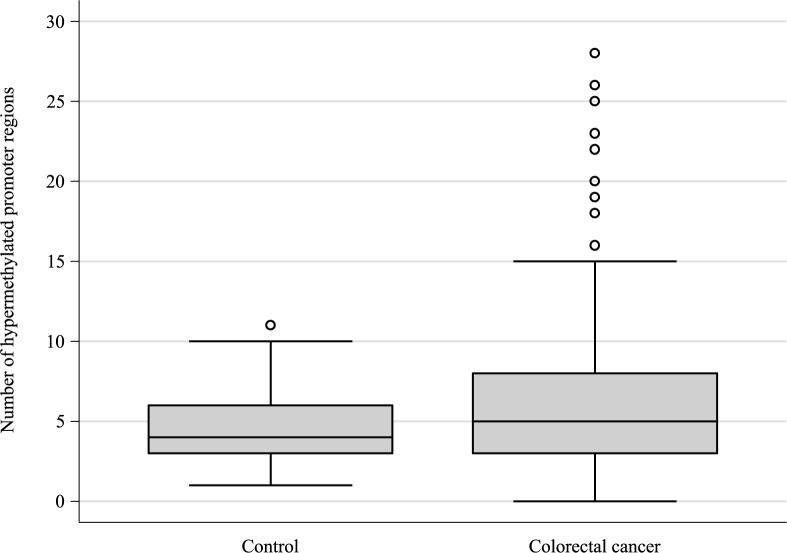
Number of methylated promoter regions according to patient group.

The hypermethylation status of all the promoter regions is provided in Table 2. The median number (range) of hypermethylated promoters were four (1–11) in the control group and five (0–28) in the CRC group (p = 0.212) (Fig 1). The median level of cell-free DNA was 4.10 ng/ml (0.31–52.19) in the control group compared with 4.00 ng/ml (0.26–132.58) in the CRC group (p = 0.982).

**Table 2:** Hypermethylation status according to gene names.

	*Colorectal Cancer (N = 193)*				*Healthy controls (N = 102)*			
	N	%	(95% CI)		N	%	(95% CI)	
*ALX4*	55	28.5	(22.2	35.4)	1	1.0	(0.0	5.3)
*APC*	81	42.0	(34.9	49.3)	33	32.4	(23.4	42.3)
*BMP3*	55	28.5	(22.2	35.4)	11	10.8	(5.5	18.5)
*BNC1*	23	11.9	(7.7	17.3)	13	12.7	(7.0	20.8)
*BRCA1*	49	25.4	(19.4	32.1)	22	21.6	(14.0	30.8)
*CDKN2A*	18	9.3	(5.6	14.3)	4	3.9	(1.1	9.7)
*HIC1*	11	5.7	(2.9	10.0)	1	1.0	(0.0	5.3)
*HLTF*	22	11.4	(7.3	16.7)	4	3.9	(1.1	9.7)
*MGMT*	11	5.7	(2.9	10.0)	1	1.0	(0.0	5.3)
*MLH1*	87	45.1	(37.9	52.4)	44	43.1	(33.4	53.3)
*NDRG4*	18	9.3	(5.6	14.3)	0	0.0	(0.0	3.6)
*NPTX2*	135	69.9	(62.9	76.3)	60	58.8	(48.6	68.5)
*NEUROG1*	40	20.7	(15.2	27.1)	20	19.6	(12.4	28.6)
*OSMR*	22	11.4	(7.3	16.7)	7	6.9	(2.8	13.6)
*PHACTR3*	28	14.5	(9.9	20.3)	6	5.9	(2.2	12.4)
*PPENK*	20	10.4	(6.4	15.6)	4	3.9	(1.1	9.7)
*RARB*	49	25.4	(19.4	32.1)	71	69.6	(59.7	78.3)
*RASSF1A*	22	11.4	(7.3	16.7)	16	15.7	(9.2	24.2)
*SDC2*	47	24.4	(18.5	31.0)	6	5.9	(2.2	12.4)
*SEPT9*	47	24.4	(18.5	31.0)	5	4.9	(1.6	11.1)
*SFRP1*	42	21.8	(16.2	28.3)	7	6.9	(2.8	13.6)
*SFRP2*	39	20.2	(14.8	26.6)	18	17.6	(10.8	26.4)
*SPG20*	30	15.5	(10.7	21.4)	12	11.8	(6.2	19.6)
*SST*	58	30.1	(23.7	37.1)	32	31.4	(22.5	41.3)
*TAC1*	102	52.8	(45.6	60.1)	48	47.1	(37.1	57.2)
*THBD*	19	9.8	(6.0	14.9)	1	1.0	(0.0	5.3)
*TFPI2*	14	7.3	(4.0	11.9)	2	2.0	(0.2	6.9)
*VIM*	34	17.6	(12.5	23.7)	12	11.8	(6.2	19.6)
*WIF1*	19	9.8	(6.0	14.9)	4	3.9	(1.1	9.7)
*WNT5A*	12	6.2	(3.3	10.6)	5	4.9	(1.6	11.1)

Note. The number (N) of patients in each patient group along with the percentages (%) of patients according to each hypermethylated promoter region along with the number (n) of patients with corresponding 95% confidence intervals (95% CI)

**Fig 2:**
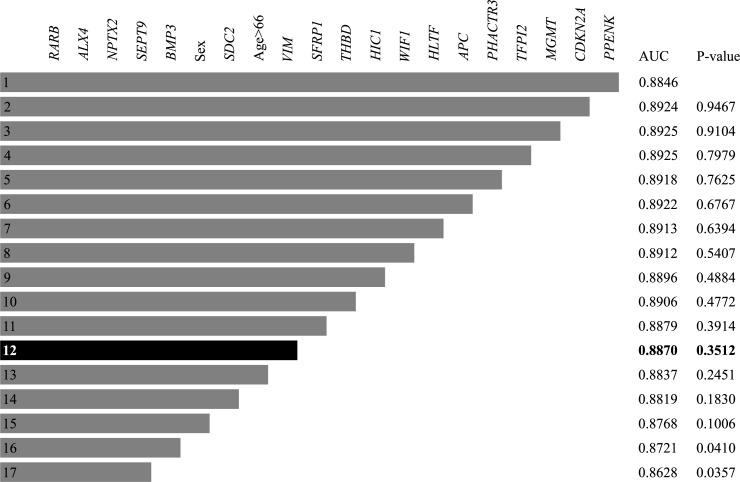
Stepwise backwards selection according to model number. Note. Logistic regression modelling with stepwise backwards selection. Potential predictor variables are located in the top row. Model number is recorded in the left column. Area under the receiver operating curve (AUC) according to model number and the P-value according to the removed predictor variable is located in the two rightward columns.

The initial univariate screening left 19 of the potential predictor variables along with sex and age>66 for further analysis. The logistic regression with stepwise backwards selection is visualised in Fig 2. Model 12 was considered the most applicable, because it contained a limited number of genes and the model did not differ from the model produced by Penalized regression using Firth’s method. Incorporation of CEA as a binary variable (>5 ng/ml) in the stepwise selection process did not lead to a difference in marker selection.

None of the prediction models showed a significant lack of model fit at the 0.05 significance level.

### Model performance

**Fig 3:**
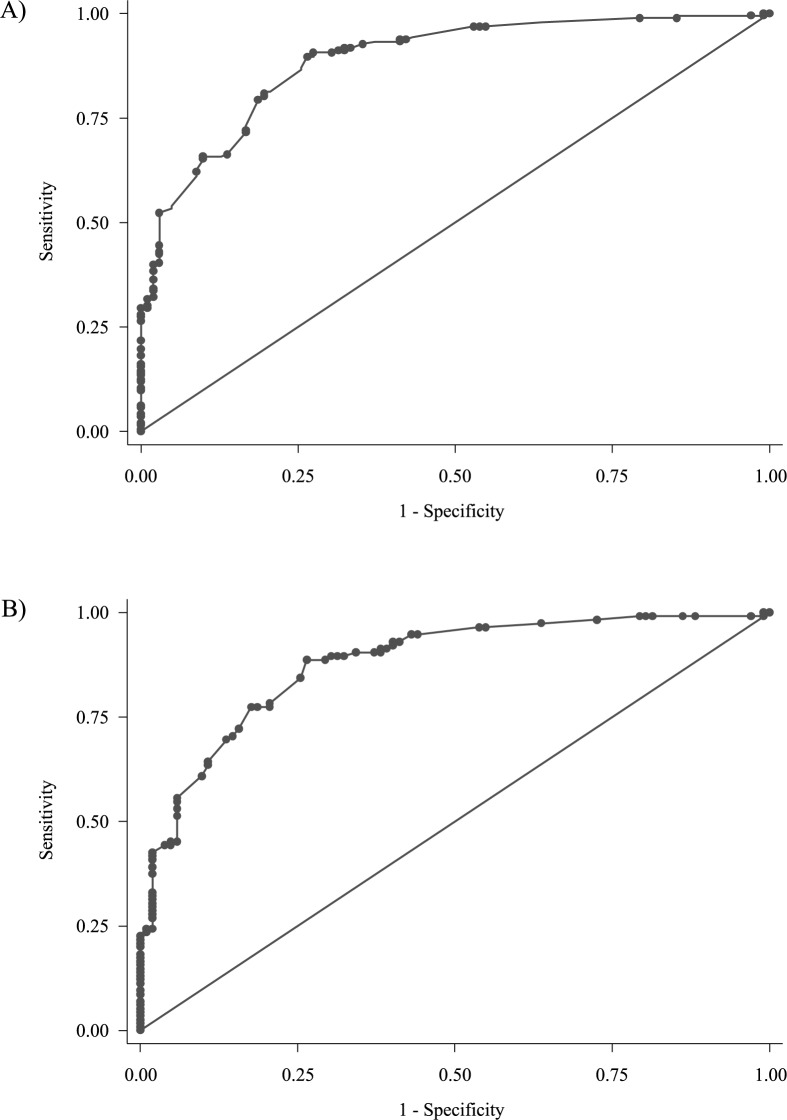
Receiver operating characteristics curves for colorectal cancer. Note. A) ROC curve for all stage CRC with a non-optimism corrected AUC of 0.8870. B) ROC curve for stage I and II CRC with a non-optimism corrected AUC of 0.8775. Receiver operating characteristics (ROC). Colorectal cancer (CRC). Area under the receiver operating characteristics curve (AUC).

Model 12 which included seven hypermethylated gene promoter regions (*ALX4*, *BMP3*, *NPTX2*, *RARB*, *SDC2*, *SEPT9*, and *VIM*) and the covariates: sex(female) and age>66 had the ability to distinguish CRC patients from patients without CRC with an optimism-corrected AUC of 0.860 (optimism = 0.027) (Fig 3A). There was a significant interaction between the prediction markers *RARB* and *VIM* (p<0.001). However, inclusion of the interaction in the model only provided modest information (optimism corrected AUC = 0.869). The interaction was therefore omitted from the final model.

Removal of the 33 patients with adenomas from the control group did not alter the efficacy of the prediction model (optimism corrected AUC = 0.863).

In order to characterise Model 12’s performance for early stage cancers, we evaluated the model by removing the patients with stage three and four CRC. This rendered an optimism-corrected AUC of 0.853 (optimism = 0.025) (Fig 3B).

The sensitivity, specificity, positive and negative predictive values of Model 12 for CRC detection in all stages and early stages only, were calculated from the ROC analysis using a 0.5 cut point value. This cut point value led to the most accurate classification of the cancer patients and the controls. The values are provided in Table 3.

**Table 3:** Model 12s sensitivity, specificity, PPV, and NPV for CRC.

	*Sensitivity*	*Specificity*	*PPV*	*NPV*
All stage CRC, %	90.7	72.5	6.0	99.8
Stage I and II CRC, %	88.7	73.5	6.1	99.7

Note: All values were calculated using the receiver operating characteristics (ROC) curve analysis at a 0.5 cut point value. The positive predictive values (PPV) and negative predictive values (NPV) are calculated from the prevalence of colorectal cancer (CRC) in the Danish population above 60 years of age (~25.000 cases in ~1.4 million people)

We also assessed the putative effect of PCR cycle numbers on the results of the assay, between CRC patients and controls. We classified the promoter regions into groups according to their cycle threshold values (0, 0–25, 25–30, and >30) (S4 Table). The distribution shows some difference in cycle threshold value between CRC patients and controls. However, because of limited power, the effect of this difference could not be evaluated in the multivariable logistic regression model.

## Discussion

In this study, we analysed a panel of 30 genes previously found to be hypermethylated in patients with CRC and other neoplasia. Through multivariable logistic regression, we selected seven gene promoter regions (*ALX4*, *BMP3*, *NPTX2*, *RARB*, *SDC2*, *SEPT9*, and *VIM*), which could distinguish early stage CRC patients from a colonoscopy verified CRC free control population.

Epi proColon^®^ (Epigenomics AG Corporation, Berlin, Germany) is the only commercially available blood based DNA hypermethylation screening test for CRC. The test is based on *SEPT9* hypermethylation, and even though there are promising results, there are still some drawbacks. In a large cross-sectional study of 1,516 patients scheduled for colonoscopy, the assay alone only reached a sensitivity of 48.2% at 95.1% specificity, showing the apparent lack of sensitivity as an individual CRC biomarker [7]. We examined the *SEPT9* v2 promoter, which is methylated in ~90% colorectal malignant and premalignant lesions [12]. Our study confirmed that hypermethylation of *SEPT9* measured in plasma is more frequent in CRC patients, but an acceptable sensitivity was only achieved with *SEPT9* included as part of a larger panel of hypermethylated promoter regions. The best individual marker was *ALX4*, however, with limited sensitivity (sensitivity of 28.5% at 99.0% specificity), further emphasising the need for a multigene hypermethylation panel for CRC detection.

Interestingly, *RARB* hypermethylation was a protective factor, more frequently hypermethylated in healthy controls (69.6%), compared to patients with CRC (25.0%). This is in contrast with the fact that *RARB* has been found to be preferentially hypermethylated in early stage rectal tumours along with high-grade cervical intraepithelial lesions and high-risk prostate cancer [13,14]. Since T3 tumours represent the bulk of our CRC cohort, along with the fact that *RARB* methylation is lost through the progression of rectal tumours, our results could indicate that *RARB* hypomethylation is a biomarker for late stage CRC [15].

We also analysed the hypermethylation frequencies of *BMP3* and *NDRG4*—the two genes implemented in the Cologuard^®^ (Exact Sciences Corporation, Madison, WI, USA) stool based CRC screening assay. *BMP3* reached our gene panel for CRC detection, and while *NDRG4* was exclusively hypermethylated in CRC patients, it was only positive in 9.3% of cases and thus, not implemented in the model. Whether this difference is due to the media of choice (blood vs. stool) or other factors remains to be elucidated. The use of stool could provide increased DNA for analysis as colonic epithelial cells undergo anoikis. Moreover, the molecular characteristics of colorectal tumour cells is influenced by their location inside the tumour—either at the luminal front or the invasion front [16,17]. The limited sensitivity of *NDRG4* in plasma could also be due to some of the factors listed in the section below.

Even though our panel of promoter regions resemble those analysed by others, there seems to be a rather large gap in both marker selection and performance. Lee et al found, that *APC*, *MGMT*, *RASSF2A*, and *WIF1* had a sensitivity of 86.5% with a specificity of 92.1% [8]. We evaluated three of these gene promoter regions, through our multivariate logistic regression. None reached the final model. The marker *SDC2* was found through an epigenome wide association study and evaluated in serum samples, revealing a sensitivity of 92.3% for stage I CRC at a specificity of 95.2% [18]. Our results on *SDC2* are more modest with a sensitivity and specificity of 24.0% and 94.1% respectively. The differences in marker performance may be due to differences in ethnicity and choice of control population. The control population in the study by Lee et al. was only specified as Asian patients undergoing a regular health check, whereas our population is from a Caucasian patient cohort of similar age and gender referred for colonoscopy with CRC symptoms.

### Limitations

There are some limitations to our analytical setup.

Hypermethylated DNA promoter regions, other than the 30 included in our initial screening panel, may be more efficient as biomarkers for CRC detection. The gene promoter regions were initially selected based on a systematic literature search [10]. We used this approach to evaluate proven CRC hypermethylation biomarkers, previously analysed in tumour remote media like blood and stool samples. Sequencing methods have since improved, providing new knowledge of complex genetic and epigenetic disruptions in colorectal carcinogenesis, and the field of epigenetic research is growing rapidly. Other approaches for the evaluation of hypermethylation biomarkers such as the implementation of an epigenome wide association study could have been used. Whether other DNA hypermethylations are more efficient as cell-free DNA based biomarkers for CRC remains to be elucidated.

Regarding the differences in marker performance, between previous studies and our current study, performance issues could be attributed to analytical limitations. Methylation specific PCR only enables the analysis of one site in each of the promoter regions, meaning that any discordance in the choice of primers and probes could lead to different results [19]. Furthermore, we used a rather small amount of plasma (<1ml) for our analysis whereas the new kit for *SEPT9* analysis (Epi proColon^®^ 2.0 [Epigenomics AG Corporation, Berlin, Germany]) isolates DNA from ~5 ml of plasma, which renders more DNA for analysis and possible replicate analysis [20]. This problem is stressed by the fact, that the number of cell-free tumour DNA fragments per 5 ml could be less than 10 for stage I CRC, making the amount of plasma used for methylation detection critical [21].

This study was exploratory in order to construct a biomarker panel of hypermethylated DNA promoter regions for CRC detection. The selection of the prediction markers was made on the entire cohort, in order to construct the most robust model. To evaluate the inherit problem of overfitting, we conducted internal validation using “leave pair out cross validation”. However, before the model can be used in a clinical setting, external validation in an independent cohort must be conducted.

### Strengths

The major strength of our study is the use of a cohort of consecutive CRC patients and a well-defined colonoscopy verified cancer-free control group with a comparable age and gender distribution. Other studies have employed less than optimal control groups, often not described in more detail than “healthy controls”. We have used patients who, albeit having symptoms, are an excellent representation of the screening population.

Another strength of our study is the method used for DNA extraction and bisulphite conversion for methylation analysis. Previously, the amount of DNA degradation through the bisulphite treatment was between 84 and 96% [22]. Through an optimized conversion step, we were able to treat the DNA with bisulphite, with a recovery as high as 60%, enabling us to detect hypermethylated DNA fragments in the limited amounts of plasma available in our study.

The development of the prediction model is in full accordance with current guidelines for the development of biomarkers for outcome detection [23].

## Conclusion

In our study, a panel of seven hypermethylated gene promoter regions (*ALX4*, *BMP3*, *NPTX2*, *RARB*, *SDC2*, *SEPT9*, and *VIM*) with the covariates: sex and age>66 was able to distinguish CRC patients from patients suspected for, but without CRC. Importantly, the model also had the ability of detecting early stage CRC with a similar performance.

This study shows, that no single hypermethylated DNA biomarker can be used in a diagnostic setting for CRC, but a panel of such may be of greater clinical interest. External validation in future studies would ensure that the diagnostic performance is reproducible.

## Supporting information

S1 Table Gene names and known function. (DOCX)

S2 Table Characteristics of gene specific primers and probes. (DOCX)

S3 Table Characteristics of the reference gene (*MEST*) primers and probes. (DOCX)

S4 Table Patients and controls according to cycle threshold. (DOCX)

S1 Fig Hypermethylation analysis of cell-free plasma derived DNA. (DOCX)

S2 Fig The association between plasma volume and cycle threshold of the reference gene. (DOCX)
