# Supplementary material for: Hypermethylated DNA, a circulating biomarker for colorectal cancer detection
Source: PLoS One. 2017 Jul 10;12(7):e0180809. doi: 10.1371/journal.pone.0180809 (PMC5507256; doi:10.1371/journal.pone.0180809)
Supplement: S1 Table — (DOCX) [file pone.0180809.s001.docx]

| **S1 Table** Gene names and known function | | | |
| --- | --- | --- | --- |
|  | *Name* |  | *Function* |
| *ALX4* | Aristaless-like homeobox 1 |  | Skull and limb development |
| *APC* | Adenomatous polyposis |  | Cellular adhesion and β-catenin regulation |
| *BMP3* | Bone morphogenetic protein 3 |  | Bone formation |
| *BNC1* | Basonuclin 1 |  | Regulates proliferation and rRNA transcription |
| *BRCA1* | Breast cancer 1 |  | DNA repair and gene transcription |
| *CDKN2A* | Cyclin dependent kinase inhibitor 2A |  | Cell-cycle regulation |
| *HIC1* | Hypermethylated in cancer 1 |  | Gene transcription and cellular division |
| *HLTF* | Helicase like transcription factor |  | Gene transcription and cellular division |
| *MGMT* | O-6-methylguanine-DNA methyltransferase |  | DNA repair |
| *MLH1* | Mutl homolog 1 |  | DNA repair |
| *NDRG4* | N-myc downstream-regulated gene 4 |  | Cell-cycle progression and regulation of mitotic signals |
| *NPTX2* | Neuronal pentraxin 2 |  | Synapse formation and non-apoptotic cell death |
| *NEUROG1* | Neurogenin 1 |  | Neuronal differentiation |
| *OSMR* | Oncostatin M receptor |  | Cytokine receptor for cell signalling |
| *PHACTR3* | Phosphatase and actin regulator 3 |  | Nuclear scaffolding of proliferating cells |
| *PPENK* | Preproenkephalin |  | Synaptic signalling |
| *RARB* | Retinoic acid receptor beta |  | Cellular signalling and retinoic acid binding |
| *RASSF1A* | Ras association domain family member 1A |  | Cell-cycle regulation and DNA repair |
| *SDC2* | Syndecan 2 |  | Cellular adhesion, signalling, and cytoskeletal structure |
| *SEPT9* | Septin 9 |  | Cell-cycle regulation and cytokinesis |
| *SFRP1* | Secreted frizzled related peptide 1 |  | Modulator of Wnt signalling |
| *SFRP2* | Secreted frizzled related peptide 2 |  | Modulator of Wnt signalling |
| *SPG20* | Spastic paraplegia 20 |  | Regulates endosomal traffic and mitochondria function |
| *SST* | Somatostatin |  | Inhibitory hormone and regulator of endocrine system |
| *TAC1* | Tachykinin precursor 1 |  | Neurotransmitting and vasodilation |
| *THBD* | Thrombomodulin |  | Inhibition of haemostasis |
| *TFPI2* | Tissue factor pathway inhibitor 2 |  | Inhibition of haemostasis |
| *VIM* | Vimentin |  | Cell shape and integrity maintenance |
| *WIF1* | Wnt inhibitory factor 1 |  | Cell fate regulation in oncogenesis and embryogenesis |
| *WNT5A* | Wnt family member 5A |  | Cell fate regulation in oncogenesis and embryogenesis |
| Note. Gene names and known function have been cross-matched with the RefSeq database (http://www.ncbi.nlm.nih.gov/refseq/) | | | |
