# Supplementary material for: Hypermethylated DNA, a circulating biomarker for colorectal cancer detection
Source: PLoS One. 2017 Jul 10;12(7):e0180809. doi: 10.1371/journal.pone.0180809 (PMC5507256; doi:10.1371/journal.pone.0180809)
Supplement: S3 Table — (DOCX) [file pone.0180809.s003.docx]

| **S3 Table** Characteristics of the reference gene (*MEST*) primers and probes | | | | | | | | |
| --- | --- | --- | --- | --- | --- | --- | --- | --- |
|  |  | *Outer primers* | | *Inner primers* | | *Probes* | *Coordinates* | *Accession no.* |
| *MEST U* | (+) | GGTTTTAAAAGTTCGGTGTTTATT | (130) | TGTTGTGGTAATTAGTATATTTT | (83) | CGCGAGTAGTTGTGTTTTGTTCGCG | 130492052 – 130492181 | NC_000007.14 |
|  | ( ̶ ) | CCIAACAACTACAACCACTCC |  | CAACCACTCCAACATACACTACA |  |  |  |  |
| *MEST M* | (+) | GCGATGGGTTTGTGCGC | (130) | CGACGTTTTAGTTTCGAGTC | (86) | CGATCGGTGGTCGGGTTCGATCG | 130486225 – 130486355 | NC_000007.14 |
|  | ( ̶ ) | GAAAAACCGATTACGCATACG |  | CGCTTCCTAAAACCAAAAATTCTCG |  |  |  |  |
| Note. The primer and probe sequences for the methylation specific polymerase chain reaction with the individual amplicon sizes represented as number of base pairs in brackets to the right of the outer/inner primers respectively. The NCBI accession number (no.) and the remapping coordinates of the amplified promoter regions are also presented. (+) Forward primer, ( ̶ ) Reverse primer. (*U*) indicates the un-methylated version of *MEST,* (*M*) indicates the methylated version of *MEST*. | | | | | | | | |
