# Supplementary material for: Hypermethylated DNA, a circulating biomarker for colorectal cancer detection
Source: PLoS One. 2017 Jul 10;12(7):e0180809. doi: 10.1371/journal.pone.0180809 (PMC5507256; doi:10.1371/journal.pone.0180809)
Supplement: S4 Table — (DOCX) [file pone.0180809.s004.docx]

| **S4 Table** Patients and controls according to cycle threshold | | | | | | | | | | | | | | | | | | | | |
| --- | --- | --- | --- | --- | --- | --- | --- | --- | --- | --- | --- | --- | --- | --- | --- | --- | --- | --- | --- | --- |
|  | *CT value: Colorectal cancer patients (N=193)* | | | | | | | | |  | | *CT value: Healthy controls (N=102)* | | | | | | | | |
|  | *No Ct* | | *0-25* | | *25-30* | | *>30* | |  | | *No CT* | | | *0-25* | | | *25-30* | | *>30* | |
|  | N | % | N | % | N | % | N | % | |  | | N | % | | N | % | N | % | N | % |
| *ALX4* | 138 | 71.5 | 8 | 4.1 | 20 | 10.4 | 27 | 14.0 | |  | | 101 | 99.0 | | 0 | 0.0 | 0 | 0.0 | 1 | 1.0 |
| *APC* | 112 | 58.0 | 25 | 13.0 | 45 | 23.3 | 11 | 5.7 | |  | | 69 | 67.6 | | 5 | 4.9 | 23 | 22.5 | 5 | 4.9 |
| *BMP3* | 138 | 71.5 | 18 | 9.3 | 21 | 10.9 | 16 | 8.3 | |  | | 91 | 89.2 | | 1 | 1.0 | 3 | 2.9 | 7 | 6.9 |
| *BNC1* | 170 | 88.1 | 4 | 2.1 | 8 | 4.1 | 11 | 5.7 | |  | | 89 | 87.3 | | 0 | 0.0 | 2 | 2.0 | 11 | 10.8 |
| *BRCA1* | 144 | 74.6 | 7 | 3.6 | 10 | 5.2 | 32 | 16.6 | |  | | 80 | 78.4 | | 3 | 2.9 | 7 | 6.9 | 12 | 11.8 |
| *CDKN2A* | 175 | 90.7 | 7 | 3.6 | 3 | 1.6 | 8 | 4.1 | |  | | 98 | 96.1 | | 0 | 0.0 | 2 | 2.0 | 2 | 2.0 |
| *HIC1* | 182 | 94.3 | 1 | 0.5 | 7 | 3.6 | 3 | 1.6 | |  | | 101 | 99.0 | | 0 | 0.0 | 0 | 0.0 | 1 | 1.0 |
| *HLTF* | 171 | 88.6 | 8 | 4.1 | 9 | 4.7 | 5 | 2.6 | |  | | 98 | 96.1 | | 1 | 1.0 | 1 | 1.0 | 2 | 2.0 |
| *MGMT* | 182 | 94.3 | 3 | 1.6 | 5 | 2.6 | 3 | 1.6 | |  | | 101 | 99.0 | | 0 | 0.0 | 0 | 0.0 | 1 | 1.0 |
| *MLH1* | 106 | 54.9 | 20 | 10.4 | 46 | 23.8 | 21 | 10.9 | |  | | 58 | 56.9 | | 2 | 2.0 | 20 | 19.6 | 22 | 21.6 |
| *NDRG4* | 175 | 90.7 | 1 | 0.5 | 1 | 0.5 | 16 | 8.3 | |  | | 102 | 100 | | 0 | 0.0 | 0 | 0.0 | 0 | 0.0 |
| *NPTX2* | 58 | 30.1 | 30 | 15.5 | 61 | 31.6 | 44 | 22.8 | |  | | 42 | 41.2 | | 5 | 4.9 | 25 | 24.5 | 30 | 29.4 |
| *NEUROG1* | 153 | 79.3 | 10 | 5.2 | 19 | 9.8 | 11 | 5.7 | |  | | 82 | 80.4 | | 0 | 0.0 | 6 | 5.9 | 14 | 13.7 |
| *OSMR* | 171 | 88.6 | 9 | 4.7 | 10 | 5.2 | 3 | 1.6 | |  | | 95 | 93.1 | | 0 | 0.0 | 2 | 2.0 | 5 | 4.9 |
| *PHACTR3* | 165 | 85.5 | 4 | 2.1 | 12 | 6.2 | 12 | 6.2 | |  | | 96 | 94.1 | | 0 | 0.0 | 1 | 1.0 | 5 | 4.9 |
| *PPENK* | 173 | 89.6 | 3 | 1.6 | 3 | 1.6 | 14 | 7.3 | |  | | 98 | 96.1 | | 0 | 0.0 | 0 | 0.0 | 4 | 3.9 |
| *RARB* | 144 | 74.6 | 8 | 4.1 | 34 | 17.6 | 7 | 3.6 | |  | | 31 | 30.4 | | 2 | 2.0 | 23 | 22.5 | 46 | 45.1 |
| *RASSF1A* | 171 | 88.6 | 10 | 5.2 | 8 | 4.1 | 4 | 2.1 | |  | | 86 | 84.3 | | 2 | 2.0 | 5 | 4.9 | 9 | 8.8 |
| *SDC2* | 146 | 75.6 | 12 | 6.2 | 26 | 13.5 | 9 | 4.7 | |  | | 96 | 94.1 | | 0 | 0.0 | 0 | 0.0 | 6 | 5.9 |
| *SEPT9* | 146 | 75.6 | 8 | 4.1 | 21 | 10.9 | 18 | 9.3 | |  | | 97 | 95.1 | | 0 | 0.0 | 0 | 0.0 | 5 | 4.9 |
| *SFRP1* | 151 | 78.2 | 12 | 6.2 | 16 | 8.3 | 14 | 7.3 | |  | | 95 | 93.1 | | 0 | 0.0 | 4 | 3.9 | 3 | 2.9 |
| *SFRP2* | 154 | 79.8 | 26 | 13.5 | 7 | 3.6 | 6 | 3.1 | |  | | 84 | 82.4 | | 5 | 4.9 | 8 | 7.8 | 5 | 4.9 |
| *SPG20* | 163 | 84.5 | 4 | 2.1 | 12 | 6.2 | 14 | 7.3 | |  | | 90 | 88.2 | | 0 | 0.0 | 0 | 0.0 | 12 | 11.8 |
| *SST* | 135 | 69.9 | 31 | 16.1 | 24 | 12.4 | 3 | 1.6 | |  | | 70 | 68.6 | | 5 | 4.9 | 23 | 22.5 | 4 | 3.9 |
| *TAC1* | 91 | 47.2 | 4 | 2.1 | 30 | 15.5 | 68 | 35.2 | |  | | 54 | 52.9 | | 0 | 0.0 | 3 | 2.9 | 45 | 44.1 |
| *THBD* | 174 | 90.2 | 4 | 2.1 | 13 | 6.7 | 2 | 1.0 | |  | | 101 | 99.0 | | 0 | 0.0 | 0 | 0.0 | 1 | 1.0 |
| *TFPI2* | 179 | 92.7 | 8 | 4.1 | 6 | 3.1 | 0 | 0.0 | |  | | 100 | 98.0 | | 0 | 0.0 | 0 | 0.0 | 2 | 2.0 |
| *VIM* | 159 | 82.4 | 6 | 3.1 | 7 | 3.6 | 21 | 10.9 | |  | | 90 | 88.2 | | 0 | 0.0 | 1 | 1.0 | 11 | 10.8 |
| *WIF1* | 174 | 90.2 | 0 | 0.0 | 4 | 2.1 | 15 | 7.8 | |  | | 98 | 96.1 | | 0 | 0.0 | 0 | 0.0 | 4 | 3.9 |
| *WNT5A* | 181 | 93.8 | 1 | 0.5 | 4 | 2.1 | 7 | 3.6 | |  | | 97 | 95.1 | | 0 | 0.0 | 0 | 0.0 | 5 | 4.9 |
| Note. The number (N) and percentages (%) of colorectal cancer patients and healthy controls according to cycle threshold value (CT) in the polymerase chain reaction. “No CT” refers to no signal. | | | | | | | | | | | | | | | | | | | | |
