# Supplementary material for: Hypermethylated DNA, a circulating biomarker for colorectal cancer detection
Source: PLoS One. 2017 Jul 10;12(7):e0180809. doi: 10.1371/journal.pone.0180809 (PMC5507256; doi:10.1371/journal.pone.0180809)
Supplement: S1 Fig — (DOCX) [file pone.0180809.s005.docx]

**S1 Fig** Hypermethylation analysis of cell-free plasma derived DNA

Note: Schematic overview of the bisulphite treatment protocol described Pedersen et al. (2012).
