# Supplementary material for: Hypermethylated DNA, a circulating biomarker for colorectal cancer detection
Source: PLoS One. 2017 Jul 10;12(7):e0180809. doi: 10.1371/journal.pone.0180809 (PMC5507256; doi:10.1371/journal.pone.0180809)
Supplement: S2 Fig — (DOCX) [file pone.0180809.s006.docx]

**S2 Fig** The association between plasma volume and cycle threshold of the reference gene

Note. Five patients did not have amplification of the reference gene (noCT). These patients were excluded from further analysis
